# Developing the science and methods of community engagement for genomic research and biobanking in Africa

**DOI:** 10.1017/gheg.2017.9

**Published:** 2017-09-04

**Authors:** P. Tindana, M. Campbell, P. Marshall, K. Littler, R. Vincent, J. Seeley, J. de Vries, D. Kamuya, the H3Africa Community Engagement Working Group

**Affiliations:** 1 Navrongo Health Research Centre, Ghana Health Service, P.O.Box 114, Navrongo, Ghana; 2 Department of Psychiatry and Mental Health, University of Cape Town, J-Block, Groote Schuur, Observatory 7925, Cape Town, South Africa; 3 Department of Bioethics, School of Medicine, Case Western Reserve University, 10900 Euclid Avenue, Cleveland, Ohio 44106-4976, USA; 4 Wellcome Trust, Gibbs Building, 215 Euston Road, London NW1 2BE, UK; 5 Freelance International Development consultant, London, UK; 6 MRC/UVRI Uganda Research Unit on AIDS, P.O. Box 49, Entebbe, Uganda; 7 Department of Medicine, University of Cape Town, Office J52-16, UCT Centre for Clinical Research, Old Main Building, Groote Schuur Hospital, Observatory, 7925 Cape Town, South Africa; 8 KEMRI/WELLCOME Trust Research Programme, Kilifi, Kenya

**Keywords:** Africa, biobanking, community engagement, evaluation, genomics

## Abstract

Historically, community engagement (CE) in research has been implemented in the fields of public health, education and agricultural development. In recent years, international discussions on the ethical and practical goals of CE have been extended to human genomic research and biobanking, particularly in the African context. While there is some consensus on the goals and value of CE generally, questions remain about the effectiveness of CE practices and how to evaluate this. Under the auspices of the Human Heredity and Health in Africa Initiative (H3Africa), the H3Africa CE working group organized a workshop in Stellenbosch, South Africa in March 2016 to explore the extent to which communities should be involved in genomic research and biobanking and to examine various methods of evaluating the effectiveness of CE. In this paper, we present the key themes that emerged from the workshop and make a case for the development of a rigorous application, evaluation and learning around approaches for CE that promote a more systematic process of engaging relevant communities. We highlight the key ways in which CE should be embedded into genomic research and biobanking projects.

## Introduction

Community engagement (CE) is gaining prominence as an important ethical requirement for genomic research and biobanking in Africa. For example, the Human Heredity and Health in Africa (H3Africa) Initiative [1] has recognised CE as one of the key elements that can support the successful implementation of genomic research on the continent [2]. H3Africa currently involves eight collaborative research projects and 18 individual research projects, which are carried out in over 20 countries in sub-Saharan Africa, four pilot biorepository research projects, and a bioinformatics network. Some of these projects involve collaborating centres across several countries in Africa while others are conducted in just one country. Most of the projects are investigating genetic and/or genomic susceptibility to specific diseases including trypanosomiasis, diabetes, HIV, tuberculosis, cardiometabolic disease, schizophrenia, cervical cancer, and rheumatic heart disease. Projects typically involve the collection of human biological materials such as blood and urine, and supporting phenotype information. These collections will be analysed for the primary research projects and most will store data and samples in repositories for future research purposes.

CE has been reinforced by the key funders of the initiative, the US National Institutes of Health and the Wellcome Trust, through increased funding to integrate CE into the next round of H3Africa projects and to explore the ethical, legal and societal implications of genomic research in Africa. Empirical studies examining CE in genomic research and biobanking are also emerging [3, 4] and there is increasing understanding that sustaining genomic research and biobanking on the continent will require effective ways of building mutually respectful and trusting relationships with relevant communities. Despite the growing interest in the concept and practice of CE, questions remain about the extent to which communities should be involved in genomic research and biobanking and how to determine the effectiveness of CE. To address these concerns, the H3Africa CE Working Group organised an evaluation workshop in Stellenbosch, South Africa, on 21st and 22nd March 2016 to provide an opportunity for all H3Africa projects to present and/or explore methods for evaluating their CE practices. In all, 34 participants from ten African countries (Botswana, Cameroon, Ethiopia, Ghana, Kenya, Mali, Nigeria, South Africa, Uganda and Zimbabwe) as well as international collaborators and CE experts from Canada, UK and USA participated in the workshop.

In this paper, we present key themes that emerged from the workshop and make a case for developing a more systematic approach to community and public engagement and its evaluation in the context of genomics and biobanking in Africa – with the promise of greater rigour, consistency and sharing of experience for improvement across engagement initiatives. We also highlight the key ways in which CE should be embedded in genomic research and biobanking projects in Africa.

## Discussion

### Why CE in genomics and biobanking?

Although CE in the context of genomic research and biobanking is relatively new, discussions at the workshop suggested that there is a strong case to be made for CE as a key component of such research particularly in the African research context. First, given growing empirical evidence that target communities may not always understand the scientific methods or the rationale for important components of genomics such as sample and data sharing, CE provides an opportunity to enhance participants’ understanding *before* the consent process. Understanding is a key element of valid consent and CE can support comprehension of the information provided. Increasingly, CE has also been described as a necessary condition for the use of broad consent for genomic research in Africa [5]. Second, CE can serve as an important tool for educating and sharing knowledge about human heredity and the synergistic effects of the environment and health with relevant groups and communities. Third, CE is not a one-way communication but an opportunity for co-learning between researchers and communities [6]. Researchers may gain a better understanding of the community's perspectives, beliefs and practices that ought to be taken into account in the general research process and in considering the disease under investigation. Communities have their own traditional explanations of the symptoms, experiences and treatments of illnesses. CE provides a platform for the negotiation of a shared understanding that draws from biomedical explanations of illness as well as more traditional and culturally sensitive explanations. Understanding a community's cultural values and potential concerns about an investigation promotes collaboration and buy-in from communities being targeted for research. This also allows researchers to be responsive to these cultural sensitivities. Such information could also play a role in the consent process to promote the ethical conduct of research. Despite this compelling rationale for CE, the workshop discussions suggested that for many of the H3Africa projects represented at the meeting, CE was an afterthought that was not planned systematically in a way that could be evaluated. This led to calls from workshop participants for a closer look at the key ways in which CE can become an integral and measurable part of the research process.

### Developing the science and methods for CE in African genomics and biobanking

An important theme that emerged from the workshop discussions was that CE should move from being a means to facilitate recruitment of participants to a more systematic process of engaging relevant communities, and in this case, on the key components of genomics and biobanking. The call for a more rigorous science of CE is not new. In 2006, Newman [7] called for devoting greater attention to developing a science of CE that can sustain research activities. Writing at the back of the closure of HIV preventive trials, he noted that ‘while millions of dollars are invested in product development, clinical training, design and building of facilities, vital processes of CE are largely [left] to trial and error’ [7]. Many lessons have been learned from the HIV trials in Africa including the importance of taking relevant communities and key stakeholders seriously. As funding for genomic research and biobanking continues to grow in Africa, it is important that communities are not left behind in the process. As scientific capacity is strengthened through increased training opportunities for African scientists and improving laboratory and research infrastructure, it is imperative that CE is also given the needed attention to support a more holistic approach to genomic research and biobanking in Africa. This would require the development of a more comprehensive approach to the planning, implementation and evaluation of CE – in other words, CE would need to become recognized as a science in its own right, drawing on the disciplines of social science and anthropology, with clear approaches, broad questions, objectives, tried and tested methodologies, sufficient funding and measurable outcomes.

But strengthening CE as research raises a number of important questions. CE as research promoted through the development and implementation of a feasible research design may lend itself to increased scientific rigor and credibility within the scientific community. Without evidence of the impact and effectiveness of these engagements, CE struggles to establish legitimacy within the broader scientific community. However, this approach holds the danger of sidelining or silencing the interaction and engagement of community members in the CE process if it adheres to a traditional hierarchy of research methods and where traditional social science disciplines are not among the most highly regarded.

Arguably, rigorous evaluation of CE needs to draw on methods appropriate for assessment of complex, dynamic multi-stakeholder processes, a number of which were explored at the workshop. Complexity sensitive evaluation approaches avoid the simple pre and post comparisons of traditional assessment and recognise that outcomes may not always be clear at the outset of a project or may change as engagement deepens and greater understanding of context develops. A range of promising evaluation approaches seek to explicitly embrace complexity while helping to identify the plausible contribution made by engagement activities. Such approaches are avowedly multi method, and emphasise rigour and triangulation [8, 9]. One challenge in developing a science of CE is establishing a comfortable space within these two extremes where CE practitioners are able to draw from the strengths of a research-orientated approach without losing the spirit of collaboration and engagement with the community. Such an approach requires clarifying the goals of the specific CE activity. This includes being clear at the onset what the goals and objectives of the engagement are and then identifying the target community and choosing CE methods and strategies carefully. Moreover, identifying dedicated and specific expertise and support systems that can ensure that CE implementation is conducted more systematically and comprehensively is necessary. Planning and integrating an evaluation component for CE is essential. Finally, successful CE depends upon dedicated funding to promote sustainable relationships with relevant communities and to support open science. We discuss each of these key elements in turn.

### Clarifying the goals of CE for genomics and biobanking in Africa

A systematic approach to CE in genomic research and biobanking in African settings requires a clear delineation of goals before embarking on any CE strategy. These goals could range from sharing information with community members and educating them about research initiatives and procedures, increasing health and research literacy including building knowledge of genetics and biobanking, to improving recruitment for a research project. Taken together, these goals promote trusted relationships between community members and investigators. Distinctions can be made between the often overlapping practical and ethical goals of CE. Some examples of practical goals may include communication and supporting the consent process while the ethical goals include trustworthiness, extending respect from individuals to communities and building legitimacy for the research project. A distinction also ought to be made between the goal of the research project and the goal of CE bearing in mind that these goals can sometimes come into conflict. There was general agreement that the goals of CE will largely depend on the nature of the research project, the context in which it is being implemented and might aim at supporting the ethical conduct of research by ensuring that the communities’ views and cultures are respected. Where CE aims to fulfill ethical goals, it might not necessarily directly support the goals of the research project. An example is where the CE goal is about ensuring that participants understand their rights about participation in research, and this includes the right to withdraw and refuse to participate based on a good understanding of the research and weighing up benefits and harms/inconveniences – whereas researchers may be interested in meeting recruitment targets within a given time frame; or where engagement means incorporating community views into the study which may change the initial research question, research design and methods. However, without the ethical goals of CE, there are no important safe-guards to hold researchers accountable to fair research practices.

It is worth noting that CE is fundamentally about building relationships [9–11]. How this relationship is built will determine the successful implementation and sustainability of genomic research projects and biobanking that incorporate CE strategies.

### Identifying target communities

The relevant groups and communities to be engaged should be identified from the onset, recognising that communities are not homogenous. For genomic research and biobanking, these groups could range from patient groups for disease specific projects to ethnic groups or ethnically mixed communities living in specific geographical settings. Identification of the target community has implications for the CE methods utilized. For example, an approach to CE for a community identified by ethnic or tribal affiliation living in geographic proximity may lead investigators to combine several CE strategies such as town hall meetings and community advisory boards. While an approach to CE that focuses on a specific disease group may include participation of the patients and family members affected by the disease, patient advocacy groups as well as treating healthcare professionals. In all cases, it is important to carefully consider who is engaged and why; and who is left out and why based on the goals and objectives of engagement.

### Choosing CE methods and strategies

Empirical evidence suggests that CE methods have been used ‘successfully’ in various research contexts in Africa [3]. Because CE is context specific, it is important that the choice of a CE method is guided by the goal of the CE, the nature of the research project and the target community to be engaged. These methods and approaches to CE may include both quantitative and qualitative research strategies. Community meetings or town hall meetings may be implemented [3]. Investigators may choose to combine focus group discussions with key informant interviews to inform the development of their CE methods. Establishing a community advisory board might be important for some projects [4]. Some researchers may consider conducting a community survey to inform the development of their CE plan. In all cases, investigators implementing CE for genomics research and biobanking in African settings must take into account traditional, political and/or administrative authority structures, where such exist and legitimately represent their ‘communities’. These may include community gatekeepers like tribal chiefs and community leaders [10, 12]. Innovativeness in the use of CE approaches, particularly those involving young people or difficult and complex topics such as genomic research and biobank, also need to be evaluated for their effectiveness. Such innovative approaches may include use of applied theatre and social media, digital storytelling and other participatory methodologies Mixed-methods can be useful to evaluate the effectiveness of the CE approach; cross triangulation of the data may also provide deeper information around the nature of research relationships e.g. the level of trust and respect that exists between research teams and target communities, and how these can be strengthened. It can also provide information about issues that are relevant and important to community members regarding the project. Information sharing is an important aspect of CE, but engagement needs to move beyond these levels to higher levels of meaningfully involvement, including opportunities for joint decision-making about research projects. The latter, while appealing, can however be fraught with many challenges; including whether researchers and research organizations would truly consider the input of community members in their research activities. Evaluating approaches used to engage communities will provide evidence on these key areas.

### Planning and integrating an evaluation component for CE

It is important that a monitoring and evaluation component is planned and integrated into the CE process, planned at the same time that the CE strategy is being planned. This ensures that there is clarity around the goals and objectives of the engagement, the appropriateness of the CE approaches and method, and of the evaluation methods that will be used and timelines. The monitoring and evaluation of the CE need to feed into the overall running of the CE strategy; the findings can be used to improve the CE activities or programmes; and to review the goals and overall focus of the CE strategy. Evaluation of a CE strategy should go beyond counting the number of people attending a CE activity or the downloads of online educational materials to a more systematic process (which can be part of monitoring); it also needs to provide explanation of what worked and what did not and the why and how. In other words, evaluation ought to measure the effectiveness and impact of the CE activity in relation to its goals and intentions, and also how the initial goals might have changed over time. Feedback sessions (of those directly involved in the engagement activities, and with key stakeholders) is an important part of monitoring and evaluation (M&E) of CE. Hence evaluation of CE needs to take account of the intentions, the processes, the outcomes and impact of the outcomes in any specific context, and in this way, can start unpacking the black box of not only what the effect of the CE strategy were, but also of the mechanisms by which the results were achieved. A good evaluation programme would be explicit that goals and CE programmes can shift dramatically, need to be flexible and accommodative of these changes.

There are several resources for evaluation of engagement projects and evaluation methods that can be useful in planning a CE evaluation. These include qualitative, quantitative and mixed methods approaches. For purposes of genomic research and biobanking, improved literacy of genomic research, particularly around the understanding of broad consent, may be an area of particular interest in CE evaluation. Similarly, the management of potential risk of stigmatization of communities targeted for genomic studies could be a focus area.

Qualitative CE evaluation strategies may include follow-up individual interviews or focus group discussions. Such activities may aim to elicit community members’ experiences and opinions about the impact and effectiveness of CE activity focused on improved genomic research literacy, or health literacy programmes that combat the negative stereotyping around particular genetic diseases such as psychiatric illnesses. Quantitative approaches may include the use of knowledge or attitude scales completed before and after a CE activity to measure change in participant perceptions, or rating scales completed after an activity to measure how effective participants found the CE activity. A mixed methods approach may be particularly helpful in contextualizing participant feedback.

However, challenges implementing successful CE evaluation include issues around feasibility. In qualitative approaches, language barriers, limited education and literacy levels amongst community members can impact the quality of feedback received about their CE experiences. Limitations with respect to quantitative approaches include concerns about the value of a single quantitative evaluation that happens immediately after a CE activity. Follow-up evaluations at 3–6 month intervals more accurately reflect the lasting impact of such activities on the lives of participants, yet reconnecting with community members after the CE event can prove challenging, compromising the response rate. Some studies have however shown that surveys designed for mobile phones can improve response rates [13].

As funders make commitments to support CE activities for genomic research and biobanking in African settings, it is important that research projects are able to demonstrate the impact of their CE strategy and justify the expenditure to funders.

### Identifying dedicated staff and funding

If CE is to be done in a more systematic way, it is important that there is dedicated staff on the research team who can devote time and efforts to support the implementation of the CE strategy. For multi-site and multi-country projects, it will be worth establishing a CE unit or team trained in CE methods and analysis within the project to coordinate CE activities across all the sites.

## Conclusion

The importance of meaningful engagement of relevant communities and stakeholders in genomic research and biobanking in Africa cannot be overemphasised. While some progress has been made to integrate CE into research projects, more needs to be done to develop the science and methods for CE that moves CE from being just a means of improving recruitment and achieving the goals of genomic research and biobanking in African settings to a systematic process of engaging relevant communities in a more meaningful and sustainable way. Developing the science and methods of CE will require commitments from research institutions, researchers, research ethics committees and funders. We have suggested some key points that need to be considered and encourage further discourse on this subject as well as empirical studies to examine effective ways in which CE can truly support ‘open’ science.
